# mGem: The complexity of viral entry—one virus, many receptors

**DOI:** 10.1128/mbio.02964-24

**Published:** 2025-02-11

**Authors:** Terence S. Dermody, Danica M. Sutherland

**Affiliations:** 1 Department of Pediatrics, University of Pittsburgh School of Medicine, Pittsburgh, Pennsylvania, USA; 2 Institute of Infection, Inflammation and Immunity, UPMC Children’s Hospital of Pittsburgh, Pittsburgh, Pennsylvania, USA; 3 Department of Microbiology and Molecular Genetics, University of Pittsburgh School of Medicine, Pittsburgh, Pennsylvania, USA; Albert Einstein College of Medicine, Bronx, New York, USA

**Keywords:** virus, receptors, neurotropism, viral pathogenesis

## Abstract

Binding to cellular receptors initiates viral replication and dictates sites in the host infected by the virus. As illustrated by mammalian orthoreovirus (reovirus), viruses can bind several types of receptors using distinct capsid components to facilitate the viral entry steps of attachment, internalization, and disassembly. The outer of the two concentric capsids of reovirus virions is formed by four viral proteins, three of which bind receptors. These capsid–receptor interactions mediate stepwise entry of reovirus, dictate viral tropism in infected animals, and expand the viral host range. Engagement of independent receptors by different capsid proteins is a property of many pathogenic viruses and illustrates common themes of receptor use in viral entry and disease.

## PERSPECTIVE

**Fig 1:**
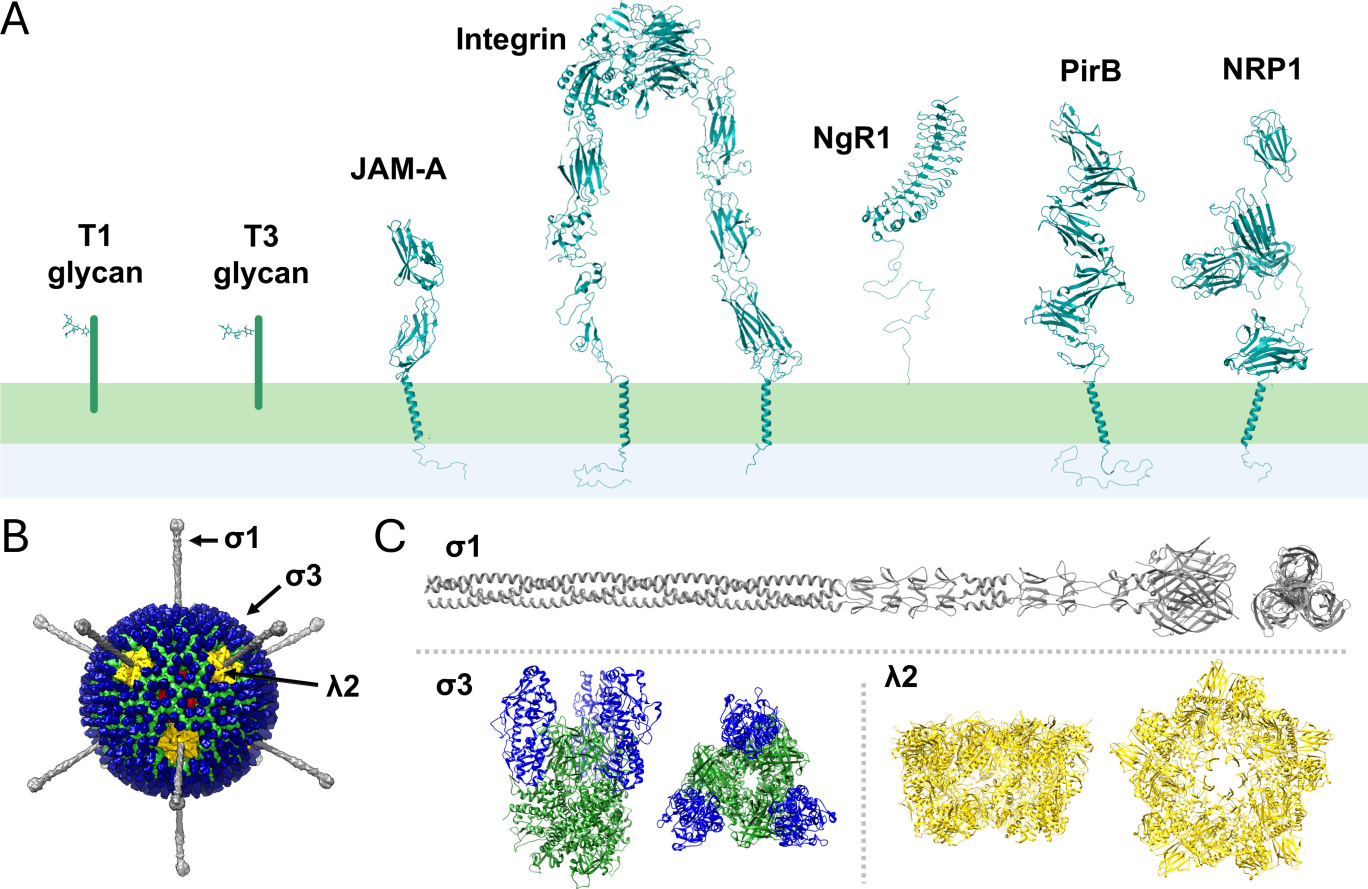
(A) Receptors bound by reovirus. Reovirus receptors modeled in stick or ribbon diagrams with ChimeraX using the following PDB IDs: 4GU3 (GM2 glycan), 3S6X (GM3 glycan), 1NBQ (JAM-A), 2VDN, 1JV2, and 2P28 (integrin), 6GRQ (PirB), and 4GZ9 and 5L73 (NRP1). Transmembrane domains are modeled using PDB ID 2K1A. (B) Model of a reovirus virion prepared using Chimera UCSF by docking σ1 crystallographic coordinates (PDB IDs: 6GAP and 3S6X) into cryo-electron microscopy image reconstruction virion coordinates (PDB ID: 2CSE), as the flexible σ1 trimer is not resolved in image reconstructions of cryo-electron micrographs of virions. (C) Reovirus receptor-binding ligands modeled in ribbon diagrams using ChimeraX and presented in side (left) and top (right) views. The σ1 trimer (gray, a.a. 27–455) was modeled by overlapping the 6GAP and 3S6X coordinates. The heterohexamer assembly of σ3 (blue) and μ1 (green) is PDB ID 1JMU, and the λ2 pentamer (yellow) was obtained from PDB ID 1EJ6.

Receptors used by viruses to bind and enter host cells serve essential roles in governing tissue tropism, pathogenesis, and host range. Viruses have adapted to use receptors that facilitate a variety of cellular functions. Virus receptors can be specialized proteins with limited tissue distribution, such as complement receptors, growth factor receptors, or neurotransmitter receptors, or more ubiquitous components of cell membranes, such as integrins and other intercellular adhesion molecules, glycosaminoglycans, or sialic acid-containing oligosaccharides (1). Viral receptors also can differ in affinity for viral attachment proteins and mediate different functions in the viral entry process. Mammalian orthoreoviruses (known colloquially as reoviruses) provide a well-established experimental system for studies of virus-receptor interactions and viral pathogenesis. Studies of reoviruses have led to the identification of seven unique receptors that can be engaged depending on the viral strain and cellular context (Fig. 1A ). What are reoviruses? And why might they use so many receptors?

Reovirus infections are common in mammals, including humans (2, 3), but disease is restricted to the very young (4–6). Although generally nonpathogenic in adults, reovirus breaks immunologic tolerance to newly introduced food antigen in mice and may trigger celiac disease in humans (7). There are three reovirus serotypes, a property determined exclusively by the σ1 viral capsid protein (8), with serotype 1 (T1) and serotype 3 (T3) strains being the best characterized. Following peroral inoculation of newborn mice, reovirus infects the intestine and displays serotype-specific patterns of systemic dissemination and tropism in the central nervous system (CNS). T1 reovirus spreads hematogenously and infects ependymal cells to cause nonlethal hydrocephalus, whereas T3 reovirus is exquisitely neurotropic, transiting efficiently within and between neurons to cause lethal encephalitis (9–12).

Reoviruses form nonenveloped, double-shelled particles that house a segmented, double-stranded RNA genome (13). The outer capsid is constructed from four viral proteins (Fig. 1B and C). The σ3 and μ1 proteins (14) form heterohexamers (σ3_3_μ1_3_) and constitute the bulk of the capsid (15). Pentamers of the λ2 protein span from the inner capsid to the outer-capsid layer at the fivefold symmetry axes (15). The σ1 protein is a filamentous trimer with head-and-tail morphology (16–18) that inserts into a turret-like opening of the λ2 pentamer (15). Remarkably, three reovirus outer-capsid proteins (σ1, σ3, and λ2) bind to the cellular receptors. The σ1 protein is the key determinant of the systemic dissemination route (19) and tropism for neural tissues (9, 10, 20), indicating a critical role for receptor recognition in dictating the pathological outcome of CNS infection.

The σ1 protein has been recognized as a reovirus attachment protein for decades, first by virtue of its capacity to bind sialylated cell-surface moieties and agglutinate erythrocytes (21, 22). T1 σ1 binds the GM2 ganglioside glycan (23), whereas T3 σ1 has more promiscuous glycan-binding behavior, engaging α2,3-, α2,6-, and α2,8-linked sialic acids (17). Engagement of these glycans contributes to reovirus-induced hydrocephalus (24) and encephalitis (25). The σ1 proteins of all three reovirus serotypes bind to junctional adhesion molecule-A (JAM-A) (26), a receptor required for hematogenous reovirus spread in mice but dispensable for CNS infection (27–29).

The σ3 protein engages at least two receptors in a serotype-independent manner: Nogo receptor 1 (NgR1) and paired immunoglobulin-like receptor B (PirB). NgR1 (30) and PirB (31) are structurally unrelated, yet they bind to the same native ligands (32) as well as the reovirus capsid protein σ3 (33, 34). Remarkably, the human, but not the murine homolog of NgR1 (29, 33), and the murine, but not the human homolog of PirB (34), serve as receptors for reovirus. Human NgR1 allows infection of nonsusceptible cells in culture (33, 35), but murine NgR1 does not bind virus efficiently and is not required for reovirus pathogenesis (29). In contrast, murine PirB promotes reovirus infection of nonsusceptible cells, but the human PirB homolog, LILRB2, does not (34). PirB is required for full reovirus neurovirulence in mice (34), indicating that more than one receptor confers reovirus CNS tropism and neurologic disease. PirB is an inhibitory immunoreceptor that, like NgR1, regulates axon growth (36). β1 integrin is a λ2 receptor required for reovirus endocytosis into some types of cells (30, 31), but functions of this receptor in reovirus disease have not been reported. In at least one case, more than a single reovirus capsid protein engages a cellular receptor. Murine neuropilin 1 (NRP1) is bound by both σ3 and λ2 and, like PirB, is required for full neurovirulence in mice (37). It is not clear how neuronal receptors that bind the same viral protein coordinate to facilitate reovirus entry. Reovirus capsid protein σ3 binds both PirB and NRP1 (along with λ2), and both receptors are required for full reovirus neurovirulence, suggesting nonredundant roles for each. The precise functions of PirB and NRP1 in reovirus infection of CNS neurons are not known, but it is possible that they contribute to infection at different sites in the brain or mediate independent steps in the reovirus entry pathway.

The multiple reovirus capsid components and host factors used as receptors appear to mediate a step-wise entry pathway for reovirus and allow infection of different cell types in the host. Initial contact between reovirus and host cells is likely to occur through low-affinity binding of σ1 to sialic acid (17, 23, 38, 39), which adheres the virus to the cell surface and induces conformational changes in σ1 (39). These conformational changes in σ1 allow the head domain to engage receptors with higher affinity to mediate essentially irreversible binding (40, 41). The exposed σ1 head domain binds JAM-A (40), which is expressed by epithelial and endothelial cells as well as by leukocytes (27). The σ1 head domain likely also binds to as yet unidentified serotype-specific receptors expressed on ependymal cells (for T1 reovirus) and neurons (for T3 reovirus) (20, 42). Outer-capsid protein σ3 appears to bind NgR1 on human neurons (33) and PirB (34) and NRP1 (37) on mouse neurons. The high avidity of σ3 binding to NgR1, PirB, or NRP1 may activate signaling to promote virus endocytosis, which has been shown experimentally for PirB (34). Interactions between λ2 and integrins also facilitate reovirus internalization by recruiting clathrin for endocytosis in at least some cell types (43–45). However, reovirus entry into neurons occurs by macropinocytosis and not clathrin-dependent uptake (46), suggesting that as-yet uncharacterized internalization receptor mechanisms function in neuronal entry. For example, it is possible that receptors in the endocytic pathway function in post-attachment disassembly steps.

Is there something unique about the capacity of reovirus to use so many receptors? Studies of many unrelated viruses, enveloped and not, indicate that the principle of multiple viral capsid components engaging discrete host receptors is common. For example, adenovirus fiber binds coxsackievirus-adenovirus receptor (CAR) (47), and the penton base binds integrins αvβ3 and αvβ5 (48); herpes simplex virus (HSV) glycoproteins gB and gC bind heparan sulfate (49–51), and gD binds herpesvirus entry mediator (HVEM/HveA) (52), nectin 1 (PRR1/HveC) (53), or nectin 2 (PRR2/HveB) (54); and human immunodeficiency virus (HIV) envelope glycoprotein gp120 apical surfaces bind CD4 (55, 56) and lateral surfaces bind chemokine receptors CXCR4 (57, 58) and CCR5 (59–61). Numerous other viruses engage glycans as attachment factors and proteinaceous moieties to facilitate high-affinity binding, internalization, or both (1). In some cases, binding to an initial receptor triggers conformational changes in the viral attachment complex that allows binding to a second receptor required for post-attachment entry steps. For example, the binding of HIV gp120 first to CD4 and then to chemokine receptors is required for the conformational changes in gp120 and gp41 to bring about fusion (62).

Why do viruses use so many receptors? Viral entry into cells is complex, and in most cases, viral entry requires virus-cell interactions in addition to those required to adhere the virus to the cell surface. Most viruses, like reovirus, adenovirus, HSV, and HIV, require additional receptors to guide post-attachment entry steps that culminate in the delivery of the viral genetic payload into the cell interior. Some of these receptors are likely used by a given virus to infect all cells susceptible to the virus, but others may function to allow the virus to infect specific cells and tissues in the host. Studies with reovirus illustrate this point. The σ1 receptor JAM-A allows the virus to infect endothelial cells in the host and disseminate hematogenously but is dispensable for infection in the CNS (27). Instead, other σ1 receptors (20) as well as those engaged by σ3 (33–35) are required for reovirus to efficiently infect neural cells. Moreover, the capacity to bind multiple receptors may expand host range of the virus and lower cross-species transmission barriers. Reovirus is a generalist pathogen capable of infecting most mammalian species. The use of highly conserved, essential receptors such as JAM-A and sialylated glycans, as well as multiple species-specific σ3 receptors, likely contributes to this property. It is possible that still other receptors are engaged to allow reovirus to infect additional host species. Thus, the variety of receptors used by viruses simplifies an understanding of viral entry into cells and explains, in part, viral tissue tropism and host range. Ongoing studies in this field will provide further insights into these important aspects of viral biology and enable more precise targeting of viruses for therapeutic purposes.
